# Clinical features and treatment for coronary sinus orifice atresia

**DOI:** 10.1186/1749-8090-10-S1-A264

**Published:** 2015-12-16

**Authors:** Naoki Tadokoro, Takaya Hoashi, Koji Kagisaki, Masatoshi Shimada, Kenichi Kurosak, Isao Shiraishi, Hajime Ichikawa

**Affiliations:** 1 Department of Pediatric Cardiovascular Surgery, National Cerebral and Cardiovascular Center, Suita, Japan; 2 Department of Pediatric Cardiology, National Cerebral and Cardiovascular Center, Suita, Japan

## Background/Introduction

Coronary sinus orifice atresia (CSOA) is a rare clinical condition, and usually does not require any treatment.

## Aims/Objectives

To review clinical features and surgical outcomes for CSOA.

## Method

From 2003 to 2015, 6 patients were diagnosed as CSOA. There were 4 females. Median age at diagnosis was 3.5 years-old (range, 0.2-73). Associated cardiac anomalies were ASD in 2 patients, functional single ventricle in 2, VSD in 1, and congenitally corrected transposition of great arteries (ccTGA) in 1. Median follow up period was 9 years (1-11).

## Results

CSOA was preoperatively diagnosed in 2 of 6 patients by late phase coronary angiography findings, intraoperatively in 3, and postoperatively in 1. Coronary venous drainage was through the left superior vena cava (LSVC) to innominate vein in 3, through Thevesian veins to right atrium in 2, and both in 1. 3 patients needed surgical treatment without any mortalities. The first patient with ccTGA had undergone the redirection of LSVC to functional left atrium at the timing of double switch operation, because postoperative transient high right SVC pressure would deteriorate coronary venous drainage, and also the presence of LSVC obstructed to create right ventricle to pulmonary artery conduit. Second patient with TA gradually developed hypoxia after the Fontan operation, which was revealed to be caused by the increase of veno-venous shunt blood flow from innominate vein to right atrium through LSVC to Thevesian veins, so that LSVC was ligated and coronary sinus was unroofed. The remaining functional single ventricle patient had undergone ligation of LSVC and unroofing coronary sinus at Fontan operation, to prevent coronary venous drainage failure.

## Discussion/Conclusion

CSOA should be suspected when LSVC drained into innominate vein existed. Surgical treatment is required if post-operative high right SVC pressure would cause coronary venous drainage failure, especially in functional single ventricle patient.

